# Supplementary material for: Eigenvalue-based entropy in directed complex networks
Source: PLoS One. 2021 Jun 21;16(6):e0251993. doi: 10.1371/journal.pone.0251993 (PMC8216510; doi:10.1371/journal.pone.0251993)
Supplement: Attachment — Submitted filename: Response to reviewer.docx [file pone.0251993.s003.docx]

**Responses to the comments**

The author thanks the editor and the reviewers for their professional and valuable suggestions and has revised the manuscript according to these suggestions, which makes the paper clearer and more readable than the original version. For each suggestion or comment, the detailed answer is given as follows:

**For Reviewer #1**

Q1: The name of the colleague or the details of the professional service that edited your manuscript.

A1: The name of the colleague or the details of the professional service that have edited in my manuscript.

 Q2: A copy of your manuscript showing your changes by either highlighting them or using track changes (uploaded as a **supporting information** file)

A2: We have carefully showing my changes by highlighting them and uploaded as a supporting information file.

 Q3: A clean copy of the edited manuscript (uploaded as the new **manuscript** file)

A3: We have a clean of the edited manuscript according to review letter.
