# Supplementary material for: Eigenvalue-based entropy in directed complex networks
Source: PLoS One. 2021 Jun 21;16(6):e0251993. doi: 10.1371/journal.pone.0251993 (PMC8216510; doi:10.1371/journal.pone.0251993)
Supplement: Attachment — Submitted filename: Response to reviewer_yansun.docx [file pone.0251993.s004.docx]

**Responses to the comments**

The author thanks the editor and the reviewers for their professional and valuable suggestions and has revised the manuscript according to these suggestions, which makes the paper clearer and more readable than the original version. For each suggestion or comment, the detailed answer is given as follows:

**For Reviewer #1**

Q1: Page 2, Line 27 "coming from ".

A1: We have rewritten as: " come from. "

Q2: Page 2, Line 34 write ``$\ldots$, which based on $\ldots$"

A2: We have carefully checked and it has revised these mistakes.

Q3: Page 3, Line 64 ``$\ldots$ and$\ldots$are$\ldots$"

A3. We have corrected these mistakes.

Q4: Page 4, Line 143 write "Definition 0.1~0.9 "

A4. We have carefully checked and corrected the "Definition 0.1~0.9 ".

Q5: Page 5, Line 124-125 There are no predicate verbs in this sentence, so it is not a complete sentence.

A5: We have rewritten the sentence and corrected these mistakes in Line 125-126.

Q6: This paper shows that “the imaginary part entropy $I(Im(A^{-}))=I(Im(L^{-}))=I(Im(Q^{-}))$”. In theory, can this precise conclusion be proved? The referee suggests them consider the theoretical proof

A6: We have given a description of the theoretical proof, and see page 7, and remark0.1 in Line 164.
